# Transcranial direct current stimulation reduces injury potential but does not enhance performance during side-cutting among individuals with chronic ankle instability

**DOI:** 10.3389/fphys.2025.1595844

**Published:** 2025-06-05

**Authors:** He Gao, Xueke Huang, Qipeng Song, Yanhao Liu, Peixin Shen, Qi Wang, Liang Zhao

**Affiliations:** ^1^ College of Sports and Health, Shandong Sport University, Jinan, Shandong, China; ^2^ Centre for Healthy Ageing and Wellness, Faculty of Health Sciences, Universiti Kebangsaan Malaysia, Kuala Lumpur, Malaysia; ^3^ Sport Science School, Beijing Sport University, Beijing, China; ^4^ China National Football Academy, Shandong Sport University, Jinan, Shandong, China

**Keywords:** tDCS, injury potential, exercise performance, functional ankle instability, unstable surface training, cutting movement

## Abstract

**Background:**

Individuals with Chronic Ankle Instability (CAI) exhibit increased injury potential and impaired movement performance, which may be associated with adaptations in the central nervous system (CNS). However, conventional CAI rehabilitation primarily concentrates on peripheral interventions, with limited emphasis on CNS-targeted therapies. Research has shown that transcranial direct current stimulation (tDCS) is a CNS intervention with the potential to improve functional deficits among individuals with CAI. This study aims to investigate the additional effects of concurrent tDCS based on Bosu ball training (BBT) on injury potential and movement performance during side-cutting among individuals with CAI.

**Methods:**

Forty participants with CAI were recruited and randomly divided into two groups, and received the tDCS + BBT or BBT interventions for 6 weeks, with three 20-min sessions per week. Before and after the intervention, kinematic and kinetic data during side-cutting were measured using a twelve-camera motion capture system and a force plate. Two-way ANOVA with repeated measures was used to analyze data.

**Results:**

Significant group-by-intervention interactions were detected in the ankle maximum inversion (p = 0.018, *η* ^2^_p_ = 0.162) and internal rotation (p = 0.023, *η* ^2^_p_ = 0.151) angles, they decreased in both groups from week 0 to week 7, and the changes were greater in the tDCS + BBT group compared to the BBT group. Significant main effects of the intervention were shown in the take-off velocity (p = 0.002, *η* ^2^_p_ = 0.271), jumping displacement (p < 0.001, *η* ^2^_p_ = 0.478), and push-off impulse (p < 0.001, *η* ^2^_p_ = 0.770), they increased in both groups from week 0 to week 7.

**Conclusion:**

Concurrent tDCS based on BBT intervention has additional effects in reducing injury potential but not in enhancing movement performance during side-cutting among individuals with CAI. Our study provides new insights for clinically reducing the injury potential among individuals with CAI.

## 1 Introduction

Ankle injuries constitute a prevalent category among sports-related injuries, accounting for roughly 40% of total occurrences (Doherty et al., 2014), with lateral ankle sprains (LAS) specifically comprising a substantial proportion of up to 80% within this category (Fong et al., 2007). Approximately 70% of individuals experiencing an acute, incident LAS may progress to develop chronic ankle instability (CAI) (Gribble et al., 2016), a condition marked by recurrent ankle sprains, diminished movement performance, decreased proprioceptive sensitivity, and persistent symptoms such as pain and inaccurate sensations (Konradsen et al., 2002; Tropp, 2002; Delahunt et al., 2010; Xue et al., 2021). Furthermore, an alarming 70% of CAI individuals exhibit degenerative changes in the ankle joint, including the development of osteoarthritis (Han et al., 2022; Zhang et al., 2023).

Notably, up to 30% of LAS occur during side-cutting maneuvers (McKay et al., 2001). Compared with individuals without CAI, those with CAI exhibit greater ankle inversion and internal rotation angles during side-cutting maneuvers (Kim et al., 2019; Simpson et al., 2020). In sports, various factors contribute to a heightened injury potential (Meeuwisse, 1991). Individuals with CAI are vulnerable to injuries, especially during side-cutting maneuvers, due to an increased tendency for ankle inversion and internal rotation (Li et al., 2019; Zhu et al., 2025). Movement performance plays a crucial in sports, as it reflects the body’s ability to meet physical demands through the coordination of take-off velocity, jump displacement, and push-off impulse (Suchomel et al., 2016). Specifically, take-off velocity reflects an individual’s agility (Morin et al., 2015); jump displacement quantifies jumping ability (Whitting et al., 2013); and push-off impulse reflects lower limb explosive strength and power output efficiency. In individuals with CAI, common performance impairments include reduced take-off velocity, shorter jump displacement, and lower push-off impulse, suggesting impaired force generation and neuromuscular control (Claudino et al., 2017). These changes directly impair functional movement abilities, making actions such as running, jumping, and rapid direction changes difficult to perform (Dos'Santos et al., 2019), which subsequently impacts daily activities (e.g., stair climbing) and sports participation. Moreover, long-term abnormal movement patterns and compensatory strategies may further contribute to secondary musculoskeletal disorders, such as knee pain and lower back pain (Terada et al., 2014).

Traditional rehabilitation programs for CAI are typically symptom-oriented and have been shown to improve issues associated with CAI, such as weak muscle strength and impaired proprioception (Alahmari et al., 2021; Wang et al., 2022). However, studies have shown that traditional interventions have limited effectiveness in reducing injury potential and improving movement performance (Han and Ricard, 2011; Hall et al., 2015). One reason for the limited clinical effects of traditional interventions may be the alterations in the central nervous system (CNS) of individuals with CAI, and this alteration may be a contributing factor to their functional impairments. The CNS changes among CAI are primarily due to peripheral mechanoreceptor damage following recurrent ankle sprains, which triggers abnormal afferent signals. These signals lead to multilevel neural adaptive reorganization through the spinal cord-cerebellum-cortex pathway, ultimately resulting in sensory-motor integration dysfunction and the consolidation of maladaptive movement patterns (Ward et al., 2015; Needle et al., 2017). Specifically, compared to those without CAI, individuals with CAI exhibited reduced excitability in the primary motor cortex (M1) (Needle et al., 2017). Pietrosimone et al. demonstrated through transcranial magnetic stimulation (TMS) that individuals with CAI exhibit significantly higher resting motor thresholds in bilateral peroneus longus muscles compared to those without CAI, suggesting a reduced cortical motor excitability among individuals with CAI (Pietrosimone and Gribble, 2012). Nanbancha et al. measured using TMS and observed that, compared to those without CAI, CAI individuals exhibited reduced excitability of the M1 (Nanbancha et al., 2019). Additionally, CAI individuals showed delayed transmission of corticospinal tract motor signals (Nanbancha et al., 2019). Therefore, there is an urgent need to develop rational and effective interventions targeting the CNS among individuals with CAI to promote functional recovery.

Transcranial direct current stimulation (tDCS), a non-invasive neuro-modulatory technique, stands as a premier approach for CNS intervention (de Moura et al., 2019). A recent study suggests that tDCS targeting the M1 may enhance cortical excitability and muscular activation among individuals with CAI, thereby facilitating motor function (Bruce et al., 2020). A key consideration in the application of tDCS is the selection of an appropriate motor task to pair with it, as the primary purpose of tDCS is to function as an adjunctive therapy to enhance task learning outcomes (Stagg and Nitsche, 2011). Bruce et al. investigated the effects of combined tDCS with eccentric ankle strength training over ten sessions during a 4-week period, which showed that the tDCS and eccentric ankle strength training intervention significantly improved cortical excitability, functional performance, and patient-reported function among individuals with CAI compared to the sham stimulation group (Bruce et al., 2020). Similarly, Ma et al. observed that tDCS combined with short-foot exercise improved dynamic balance and proprioception among individuals with CAI (Ma et al., 2020). However, ankle sprains typically occur during physical activity or under conditions of postural instability, whereas most previous studies have combined tDCS with motor tasks performed in a stable body position. Therefore, combining tDCS with unstable surface training, such as Bosu ball training (BBT), may yield better outcomes in improving functional impairments associated with CAI. In addition, studies have found that BBT is effective in reducing the injury potential among individuals with CAI. A prospective controlled study conducted by Verhagen et al. demonstrated that BBT could significantly reduce the incidence of ankle sprains in volleyball players (Verhagen et al., 2004). Sepasgozar et al. also found that BBT could improve the postural control during jumping and landing among individuals with CAI (Sepasgozar Sarkhosh et al., 2024). Huang et al. demonstrated that a 6-week combined intervention of tDCS and BBT was more effective than BBT alone in reducing injury potential after drop-landing on a simulated ankle inversion device among individuals with CAI (Huang et al., 2024). However, drop-landing may not fully replicate the complex, multi-directional forces experienced in real-world scenarios. Side cutting requires the individual to quickly change direction, which places a greater emphasis on the ankle’s ability to provide stability during rapid lateral movements, which may better simulate the dynamic movements and stresses encountered during sports and daily activities, providing a more comprehensive assessment of ankle injury potential.

The effects of tDCS on injury potential and movement performance during side-cutting among individuals with CAI remain unclear. The purpose of this study is to investigate the additional effects of concurrent tDCS based on BBT on injury potential and movement performance during side-cutting among individuals with CAI. We hypothesize that ① both the tDCS + BBT and the BBT interventions will reduce ankle inversion and internal rotation angles during side-cutting, and the decreases will be greater with the tDCS + BBT intervention compared to the BBT intervention alone; ② both the tDCS + BBT and the BBT interventions will increase take-off velocity, jumping displacement and push-off impulse, and the increases will be greater with the tDCS + BBT intervention.

## 2 Materials and methods

This study was conducted in strict accordance with the CONSORT-Outcomes 2022 Extension to ensure the standardization and accuracy of the reporting of research results (Butcher et al., 2022).

### 2.1 Participants

An *a priori* power analysis (G*Power Version 3.1) indicated that a total of at least 28 participants was needed to obtain an alpha level of 0.05 and a statistical power of 0.95 based on a previous report, which compared the peak ankle inversion angular velocity before and after tDCS + BBT vs. BBT interventions among individuals with CAI (*η* ^2^_p_ = 0.118) (Huang et al., 2024). Considering the potential sample loss, a total of 40 individuals with CAI were recruited for this study.

Utilizing posters and electronic flyers as recruitment strategies, a comprehensive eligibility assessment was conducted on 75 potential participants from a local university. Adherence to the International Ankle Consortium guidelines (Gribble et al., 2016) ensured rigorous inclusion and exclusion criteria were established. The inclusion criteria encompassed: ① experiencing at least one severe ankle sprain within the past year, accompanied by symptoms like pain, swelling, and an inability to engage in normal activities for over a day; ② encountering at least two episodes of ankle “giving way” in the preceding 6 months; ③ persistent sensations of ankle instability and functional limitations during daily activities; ④ achieving a Cumberland Ankle Instability Tool (CAIT) score below 24 points. Conversely, exclusion criteria excluded individuals with: ① a history of lower limb fractures or surgical interventions; ② acute injuries, such as sprains, to the lower limbs within the last 3 months; ③ bilateral chronic ankle instability (CAI); ④ a history of epilepsy.

**FIGURE 1:**
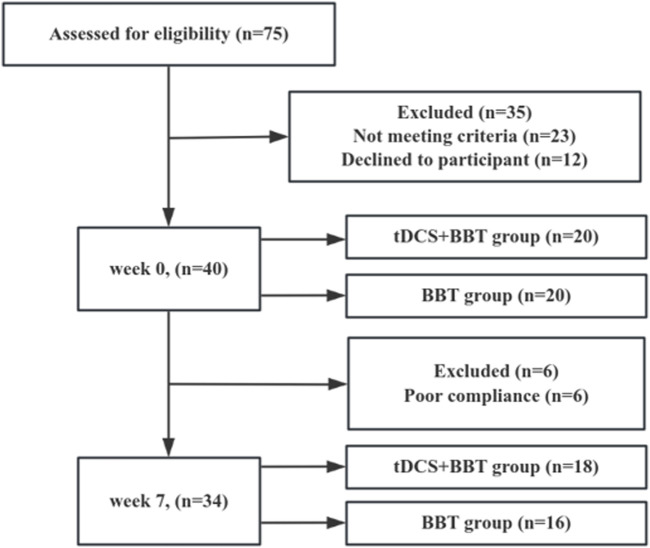
Participant flow chart. A flow chart depicting participation from week 0 to week 7 indicates that the final analysis encompassed data from 34 participants. Out of the original 75 participants assessed, 41 were excluded for various reasons.

After a meticulous eligibility assessment, 40 participants with CAI were recruited. Subsequently, the simple randomization grouping method was adopted. The participants were numbered from 1 to 40 according to the recruitment order, and random numbers were generated through Excel software to divide them into the tDCS + BBT group and the BBT group. Participants in the tDCS + BBT group underwent concurrent tDCS therapy and BBT, whereas participants in the BBT group received sham tDCS during their BBT sessions, spanning a 6-week period. Each week comprised three sessions of 20-min (Huang et al., 2024) (Figure 1). No statistically significant differences were observed in age, body height, body mass, or CAIT scores between the two groups. Prior to participation, all participants provided their written informed consent, which had been approved. Human participation in this study was granted ethical approval by the Ethics Committee of Exercise Science, Shandong Sport University (approval number: 2022047), and adhered to the principles outlined in the Declaration of Helsinki.

### 2.2 Protocol

#### 2.2.1 Bosu ball training (BBT)

Before performing BBT (Bosu ball, Yottoy, 58 cm diameter, China), participants were instructed to complete a 10-min warm-up, which included single-leg static standing and single-leg vertical jumps. The warm-up was specifically targeted at the body parts that would be involved in the subsequent training.

**FIGURE 2:**
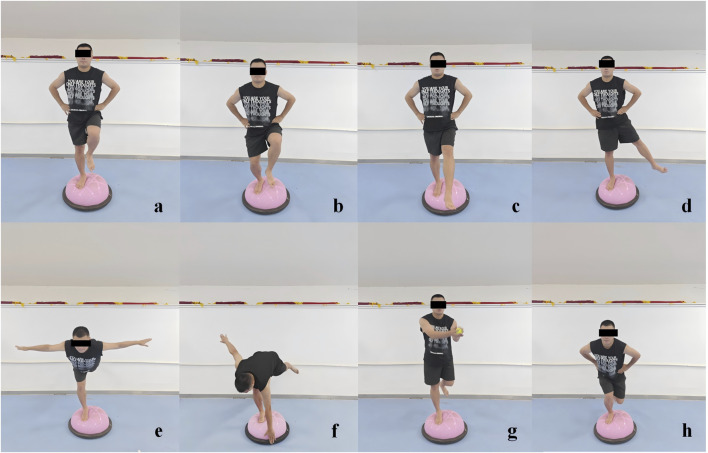
Illustrations of the Bosu ball training movements. **(a)** Single-leg stance, **(b)** single-leg squat, **(c)** single-leg stance with forward-backward leg swing, **(d)** single-leg stance with medial-lateral leg swing, **(e)** swallow balanced stance, **(f)** bending over to touch the edge while single-leg stance. **(g)** Catching a ball while single-leg stance, and **(h)** single-legged squat take-ups.

The participants engaged in a structured, progressive training program for the BBT in a barefoot condition. The initial 2 weeks focused on foundational exercises, including single-leg stance, single-leg stance with forward-backward leg swing (within a 30°–45° range), single-leg stance with medial-lateral leg swing (within a 20°–30° range), and single-leg squats. In the subsequent 2-week period (weeks 3–4), the program escalated in complexity, incorporating the swallow balanced stance (The swallow balanced stance is a dynamic one-leg support balance posture, characterized by a stable supporting leg, with the other leg and upper body forming a horizontal line, simulating the posture of a swan spreading its wings during flight), as well as increasing the range of motion for the forward-backward and medial-lateral leg swings to 45°–60° and 30°–45° respectively, and incorporating single-legged squat take-ups. During the final 2 weeks (weeks 5–6), the program further intensified, introducing the additional challenge of catching a ball while maintaining single-leg stance, and integrating bending over to touch the edge of the BBT while balancing on one leg, alongside the previously established exercises with enhanced leg swing ranges (Huang et al., 2024) (Figure 2). To prevent participants from being injured during the training (such as falling or spraining an ankle), we have arranged for testers to provide protection around them during the BBT training. Additionally, we have placed support bars in front of the Bosu ball and informed the participants that they can grab the support bars to stabilize their bodies in case of instability.

Each movement was executed for a duration of 30 s, with each exercise repeated five times, interspersed with a 30-s rest period between each movement. The cumulative duration of each training session approximated 20-min.

#### 2.2.2 Transcranial direct current stimulation (tDCS)

**FIGURE 3:**
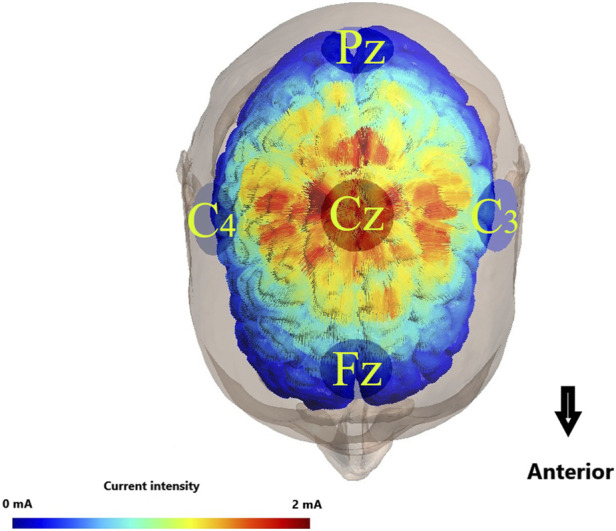
Illustration of transcranial direct current stimulation electrode placemen. Colors with warmer shades indicate the larger value of the modeled electric field normal component, and colors with cooler shades represent the smaller value of the same component.

An advanced 8-channel high-precision transcranial direct current stimulation (StarStim8, NE transcranial direct current stimulator, Spain) was utilized for the administration of direct current stimulation to the CNS during the BBT. Prior to application, the conductive electrode pads were saturated with a 0.9% saline solution to ensure optimal conductivity. The input electrode, serving as the anode, was strategically positioned over the M1 region (Cz), adhering to the internationally recognized 10–20 electrode placement system. Subsequently, four return electrodes were systematically placed at Fz, C4, Pz, and C3, each maintaining a standardized distance of 7.5 cm from the Cz area (Villamar et al., 2013) (Figure 3).

For the tDCS + BBT group, the stimulation protocol commenced with a gradual increase in current from 0 mA to 2 mA over a period of 30 s, maintaining this intensity for a duration of 19-min to ensure optimal therapeutic effect. Following this, the current was gradually reduced back to 0 mA over another 30-s period. In contrast, for the BBT group, a sham tDCS was delivered. While the current initially ramped up to 2 mA within 30 s, it was immediately reduced to 0 mA and remained at this level for the remainder of the session, acknowledging that sensations associated with direct current, such as itching or mild discomfort, typically subside within the initial 30 s of tDCS application, thereby preserving the integrity of the single-blind study design (Nitsche et al., 2003; Paulus, 2003).

### 2.3 Side-cutting test

Prior to the commencement of the side-cutting test, participants conducted a structured warm-up routine consisting of 10-min of jogging and stretching. Following the warm-up, each participant executed five consecutive side-cutting trials, with the aim of mitigating the potential influence of learning effects on the results.

**FIGURE 4:**
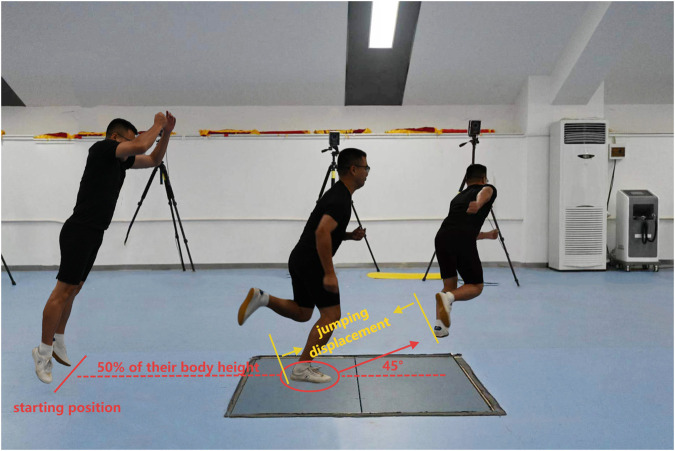
Side-cutting test. Participants stood at 50% body height from a force platform’s center, executed a double-leg jump landing on a 15 cm circle, then performed a 45-degree side-cutting jump for max distance, followed by a 3-m sprint.

The participants stood at a standardized distance of 50% of their body height from the central position of a force platform (AMTI, AMTI Inc., Watertown, MA, United States). Upon receiving a cue from the tester, the participants executed a double-leg jump forward and upward, landing with their test leg accurately positioned within a circle (15 cm in diameter) marked on the force platform. Immediately after landing, they performed a maximum 45-degree side-cutting jump to achieve the farthest possible distance, followed by a sprint over a distance of approximately 3 m. The cutting angle was marked on the ground with tape to visually represent the proper 45° angle that participants were required to make. Five side-cutting trials were recorded for each participant at both week 0 and week 7 of the study. To ensure optimal recovery and prevent fatigue, a minimum rest period of 1 min was allowed between consecutive trials (Figure 4).

Forty-three reflective markers were meticulously positioned on the participants’ bony landmarks to ensure accurate tracking during data acquisition. Utilizing a motion analysis system equipped with twelve high-speed cameras at 200 Hz (Vicon, Oxford Metrics Ltd., United Kingdom) and the force platform at 1,000 Hz, comprehensive kinematic and kinetic data were collected throughout the test.

### 2.4 Data reduction

The determination of initial contact was achieved by identifying the instant when the vertical ground reaction force (GRF) surpassed a threshold of 5 N, marking the commencement of the side-cutting process. Similarly, the endpoint was established when the vertical GRF declined below 5 N, signifying the cessation of force transmission between the test leg and the force plate. The raw kinematic and kinetic data were imported into the V3D software (C-Motion, Germantown, MD, United States). Kinematic data were filtered using a fourth-order low-pass Butterworth filter with a cut-off frequency of 15 Hz (O'Connor and Bottum, 2009). A cut-off frequency of 100 Hz was applied to the kinetic data (Nigg et al., 2009), which was normalized to body mass.

### 2.5 Variables

The period between 130–180 ms following a side-cutting foot strike is particularly susceptible to ankle injuries (Parenteau et al., 1998; Kristianslund et al., 2011). Therefore, we measured the maximum inversion and internal rotation angles of the ankle joint during this phase. The ankle inversion angle was defined as the maximum Euler angle of the foot relative to the tibia in the coronal plane, the ankle internal rotation angle was defined as the maximum Euler angle of the foot relative to the tibia in the horizontal plane, from the initial contact to the endpoint. Take-off velocity was defined as the velocity of the body’s center of mass at the moment when the heel of the testing foot left the force plate, reflecting an individual’s agility (Morin et al., 2015). Jumping displacement was defined as the distance between the coordinates of the heel marker of the test foot at the moment of contact with the force plate and the coordinates of the heel marker of the opposite foot at the moment of landing. This measure was used to quantify jump performance (Whitting et al., 2013). Push-off impulse (Im) was defined as the force-time integral during the side-cutting push-off process (Cabarkapa et al., 2023). This parameter is also a critical factor affecting jump performance (Lis et al., 2022). It was calculated as the area under the force-time curve in the anterior-posterior (XIm), medial-lateral (YIm), and vertical directions (ZIm) during the side-cutting push-off phase, by the following formula:

$$\text{Im}={\left[\left({XIm}^{2}+{YIm}^{2}+{ZIm}^{2}\right)\right]}^{0.5}$$

The push-off phase was defined as the period from the second rise of the vertical GRF to the moment when the toe of the testing foot leaves the ground.

### 2.6 Statistical analysis

Statistical analysis was performed using SPSS (26.0, IBM, New York, United States). The Shapiro-Wilk test was used to examine the normality of the data distribution. A two-way analysis of variance with repeated measures was used to determine the effects of intervention and grouping, and their interactions on each of the outcome variables. A Bonferroni-adjusted *post hoc* analysis was conducted when intervention-group interaction was detected. The Partial eta squared (*η* ^2^_p_) was used to represent the effect size of the main or interaction effects in the two-way repeated measures analysis of variance, which can reflect the proportion of the total variability in the outcome that can be explained by a particular factor (e.g., intervention, group, or their interaction). The thresholds for Partial eta squared were as follows: 0.01–0.06 = small; 0.06–0.14 = medium; >0.14 = large (Pierce Ca and Aguinis, 2004). Cohen’s *d* was used to represent the effect size of the pairwise comparisons in the post-hoc analysis. The thresholds for Cohen’s *d* were as follows: 0.20–0.50 = small, 0.50–0.80 = medium, >0.80 = large (Cohen, 2016). The significance level was set at p < 0.05.

## 3 Results

The Shapiro-Wilk test confirmed the normal distribution of all dependent variables. According to the pre-set exclusion criteria, six participants were excluded from the final analysis due to time-schedule conflicts, which led to insufficient intervention compliance (with a training completion rate of less than 80%). Consequently, 18 participants in the tDCS + BBT group and 16 in the BBT group completed the post-intervention tests (Figure 1).

**FIGURE 5:**
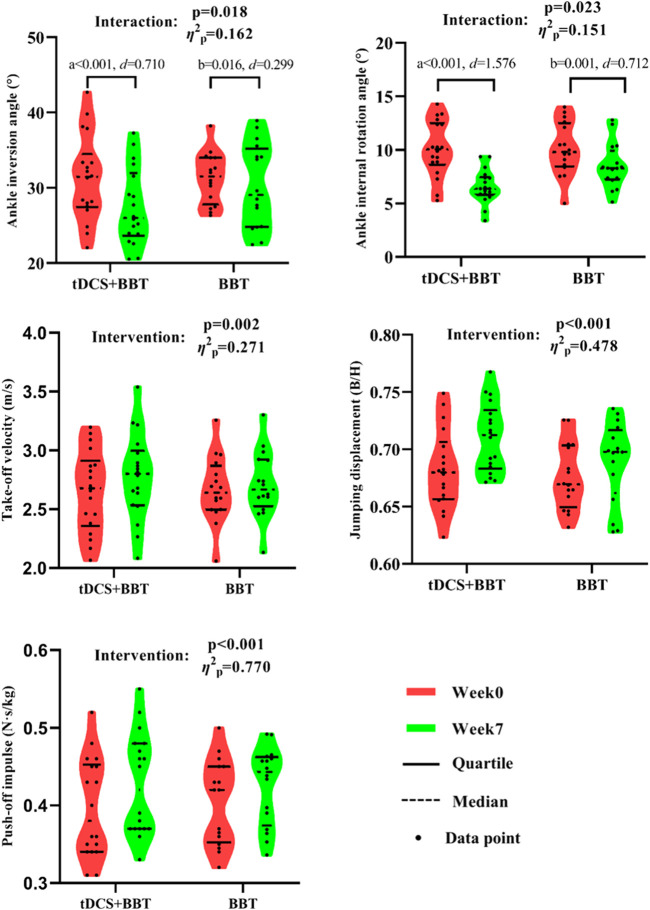
Kinematic and kinetic data between the tDCS + BBT group and the BBT group before and after intervention. a: Significant difference compared with week 7 in tDCS + BBT group; b: Significant difference compared with week 7 in BBT group. tDCS: Transcranial direct current stimulation; BBT: Bosu ball training.

Figure 5 presents the descriptive statistics and subgroup comparisons for the variables. Significant intervention-by-group interactions were observed for both ankle inversion (p = 0.018, *η* ^2^_p_ = 0.162, large effect) and internal rotation (p = 0.023, *η* ^2^_p_ = 0.151, large effect) angles, indicating differential responses to the interventions. From week 0 to week 7, significant decreases were found in both angles within the tDCS + BBT group (ankle inversion: p < 0.001, *d* = 0.710, medium effect; internal rotation: p < 0.001, *d* = 1.576, large effect), and within the BBT group (ankle inversion: p = 0.016, *d* = 0.299, small effect; internal rotation: p = 0.001, *d* = 0.712, medium effect). At week 7, the internal rotation angle of the tDCS + BBT group was significantly smaller than that of the BBT group (p = 0.004, *d* = 1.035, large effect), suggesting a greater reduction in internal rotation in the combined intervention group. Furthermore, significant main effects of the interventions were detected on take-off velocity (p = 0.002, *η* ^2^_p_ = 0.271, large effect), jumping displacement (p < 0.001, *η* ^2^_p_ = 0.478, large effect), and push-off impulse (p < 0.001, *η* ^2^_p_ = 0.770, large effect). These variables increased in both groups from week 0 to week 7, suggesting overall improvements across both groups.

## 4 Discussion

The results supported hypothesis #1 and rejected hypothesis #2. Specifically, the combination of tDCS and BBT intervention demonstrated greater efficacy than BBT alone in reducing maximum ankle inversion and internal rotation angles, indicating a decrease in injury potential. Furthermore, both groups exhibited comparable improvements in take-off velocity, jumping displacement, and push-off impulse, emphasizing an overall enhancement in movement performance across both interventions.

### 4.1 The effects of BBT training on side-cutting in CAIs

Our findings indicate that BBT significantly reduces the maximum angles of ankle inversion and internal rotation, while also enhancing take-off velocity, jumping displacement, and push-off impulse during the side-cutting jump step. We speculate that BBT may lower the injury potential during side-cutting and improve movement performance among individuals with CAI. The previous studies confirmed our speculation. Verhagen et al. conducted a prospective controlled study involving 116 volleyball teams (1,127 players). The intervention group underwent BBT training, while the control group received standard training. Over the 36-week period, the intervention group showed a significant reduction in the incidence of ankle sprains (Verhagen et al., 2004). Wester et al. conducted a study in which 48 individuals with CAI were divided into a BBT group and a control group. The participants underwent 12 weeks of BBT training or conventional training respectively. Follow-up over 230 days after the intervention revealed that the recurrence rate of ankle sprains was significantly lower in the BBT training group compared to the control group, and there was a notable reduction in the number of individuals with ankle instability (Wester et al., 1996). Nam et al. observed that BBT could bring about positive changes in the gait velocity and balance ability of healthy adults (Nam et al., 2016). Furthermore, BBT interventions have been proven beneficial for older adults, which could significantly improve dynamic balance and reduce fall concerns (Pirauá et al., 2019). However, our results diverge from those reported by Cressey et al., who caution against applying unstable surface training to elite athletes due to potential variability in responses based on population characteristics (Cressey et al., 2007). This discrepancy may stem from the differing functional capabilities between CAI individuals and elite athletes, with the latter potentially experiencing a ceiling effect during training.

The mechanism by which BBT reduces injury potential and enhances movement performance may be attributed to its improvement of proprioception and muscle strength around the trunk and ankle joints. Studies have indicated that BBT could enhance proprioception around the trunk and ankle joints, enabling individuals with CAI to better perceive the position of the ankle joint during side-cutting and make timely adjustments to prevent injury (Alizamani et al., 2023). Additionally, BBT could improve muscle strength around the trunk and ankle joints (Cuğ et al., 2016; Gibbons and Bird, 2019), with weaker trunk muscle strength being associated with an increased lower limb injury potential, particularly in activities involving jumping, leaping, and rapid running. Moreover, Bouillon et al. observed that BBT could activate the peroneus longus (Bouillon et al., 2020), which responds defensively to sudden ankle inversion, helping to prevent LAS (Tashiro et al., 2021). The improvement in both muscle strength and proprioception of the trunk and ankle joints also contributes to maintaining optimal posture during physical activity, reducing unnecessary energy expenditure (Liu et al., 2024), enhancing the efficiency of force transmission between the limbs, and improving overall body coordination, thereby leading to enhanced movement performance. Additionally, BBT can increase muscle activation, enhance the maximum strength and explosive power of the lower limbs, and consequently improve movement performance (Kohler et al., 2010; Hall et al., 2018).

### 4.2 The additional effects of tDCS combined with BBT intervention in CAIs

Our study has revealed that the combined intervention of tDCS with BBT exhibits greater effectiveness in reducing the maximum angles of ankle inversion and internal rotation among individuals with CAI compared to BBT alone. Therefore, we speculate that the combination of tDCS and BBT is more effective in reducing the injury potential among individuals with CAI. Notably, there is a dearth of previous studies specifically investigating the impact of tDCS on injury potential in this population. Some previous studies shown that tDCS, when combined with functional training, could elicit additional benefits (other than injury potential) among individuals with CAI, as well as among individuals without CAI and older adults. Bruce et al. observed that a program combining tDCS with eccentric training led to improved cortical excitability, functional performance, and patient-reported outcomes among individuals with CAI (Bruce et al., 2020). Notably, these improvements were not sustained in the eccentric training-only group, emphasizing the added value of tDCS.

The mechanism of the additional effects of tDCS may be attributed to the following two aspects: Firstly, the M1 plays a pivotal role in motor learning and consolidation (Muellbacher et al., 2002) making it an attractive target for motor rehabilitation and motor learning (Reis and Fritsch, 2011). By modulating M1 excitability with tDCS, which may help to facilitate the recruitment of neurons essential for motor skill acquisition, thereby optimizing the effectiveness of training. In addition, tDCS promotes neural plasticity by regulating synaptic connections and functions, thereby enhancing motor learning (Madhavan and Shah, 2012) In our study, participants performed functional training on an unstable surface, requiring continuous ankle adjustments to maintain stability. We deduced that concurrent M1 tDCS could enhance this learning process, and this effect was consolidated over the 6-week intervention period, leading to a reduced injury potential. Previous research has also demonstrated that tDCS can facilitate motor learning and the long-term consolidation of skills (Reis et al., 2009; Stagg et al., 2011).

Our results indicate that tDCS combined with the BBT did not yield additional benefits in enhancing movement performance compared to BBT alone among individuals with CAI. Notably, most previous studies have focused the effect of tDCS specifically on movement performance on healthy individuals rather than CAI. Hu conducted a systematic review aimed at exploring the effects of tDCS on upper limb muscle strength and endurance in healthy participants. The review found that tDCS had no significant effect on improving upper limb muscle strength, but it significantly enhanced upper limb endurance performance, particularly on the non-dominant side (Hu et al., 2022). Similarly, Romero et al. reported that tDCS does not improve jump performance in young healthy men (Romero-Arenas et al., 2021). Shyamali suggested that the effects of tDCS on performance may depend on the type of task. They conducted a meta-analysis and found that tDCS can improve endurance performance in cyclists, but it does not enhance sprint performance (Shyamali Kaushalya et al., 2022).

The observed lack of additional benefits in improving movement performance with tDCS can be attributed to its uncertain effectiveness in enhancing the explosive power of lower limb muscles. Previous research has demonstrated that tDCS does not augment maximum force and explosive power (Alix-Fages et al., 2019; Holgado et al., 2019). Since these factors are directly associated with take-off velocity, jumping displacement, and push-off impulse during side-cutting movements (Peterson et al., 2006; Barr et al., 2014; Hertel and Corbett, 2019), this provides a plausible explanation for our result. In addition, several studies have proven that there is a positive correlation between the effects of tDCS and the intervention period (Talimkhani et al., 2019). However, due to the relatively short training period (6 weeks) in our study, the improvement in movement performance by tDCS was not satisfactory.

### 4.3 Clinical implications

To facilitate clinical translation of our findings, we propose the following evidence-based recommendations for implementing tDCS and BBT interventions in rehabilitation programs targeting CAI. Our findings indicate that distinct therapeutic strategies should be adopted for individuals with CAI based on different treatment objectives: For reducing injury potential, we recommend combining tDCS (2 mA over M1 cortex) with BBT for 3 sessions/week over 6 weeks, as this protocol significantly reduced the maximum ankle inversion and internal rotation angles. For enhancing movement performance, BBT alone (same frequency/duration) is sufficient, as tDCS provided no additional benefit. However, as the underlying mechanisms of tDCS remain unclear, it currently does not meet the requirements for widespread clinical application in this field. Further research is needed to establish standardized protocols and to determine long-term effects. In addition, when applying tDCS clinically, it is necessary to comprehensively understand the patient’s condition. For individuals with conditions such as metallic implants in the skull, epilepsy, and severe heart disease, the use of tDCS is prohibited. Future research should involve larger sample sizes to further investigate the underlying mechanisms of tDCS and BBT in treating functional impairments among individuals with CAI. Additionally, follow-up assessments following the intervention should be conducted to provide a more robust theoretical foundation for the widespread application of combined tDCS and BBT treatments in clinical practice. Future studies are encouraged to compare outcomes across different types of athletes to determine whether sport-specific demands influence the effectiveness of these interventions. Additionally, studies using functional near-infrared spectroscopy and functional magnetic resonance imaging have found that, compared to individuals without CAI, those with CAI exhibit greater standard deviations in oxygenated hemoglobin levels in the supplementary motor area. Furthermore, activation changes were observed in the ipsilateral superior temporal gyrus, precuneus, supplementary motor area, superior frontal gyrus, and contralateral postcentral gyrus (Rosen et al., 2019; Li et al., 2024). Future research could further apply tDCS to stimulate different brain areas, and compare the resulting functional improvements.

### 4.4 Limitations

Our study has several limitations. Firstly, although the sample size (n = 34) was determined based on calculations from previous similar studies, it remains relatively small, which may limit the generalizability of the findings. Secondly, no follow-up assessment was conducted after the 6-week intervention, leaving the long-term effects of the intervention uncertain. Thirdly, there is a lack of in-depth exploration of the mechanisms of tDCS and BBT; we remain unclear about how tDCS reduces injury potential and improves movement performance, and the study does not explore the mechanisms through which BBT enhances performance and reduces injury potential. Fourthly, the study did not incorporate self-reported outcome measures from individuals with CAI, making it difficult to gauge the extent of improvement in their actual lives due to the intervention. Fifthly, as tDCS was administered in combination with BBT, the effect of tDCS alone in treating individuals with CAI remains unclear. However, the primary purpose of incorporating tDCS was to serve as an adjunctive therapy aimed at enhancing the efficacy of task-specific motor learning rather than functioning as a sole intervention. Sixth, the study lacks individualized stimulation guided by neuroimaging.

## 5 Conclusion

Compared with the BBT, the tDCS combined with the BBT intervention was more effective in reducing injury potential, but did not yield any additional benefits in enhancing movement performance during side-cutting among individuals with CAI. Our study provides new insights for clinically reducing the injury potential among individuals with CAI.

## Data Availability

The raw data supporting the conclusions of this article will be made available by the authors, without undue reservation.

## References

[B1] Alahmari K. A. Kakaraparthi V. N. Reddy R. S. Silvian P. Tedla J. S. Rengaramanujam K. (2021). Combined effects of strengthening and proprioceptive training on stability, balance, and proprioception among subjects with chronic ankle instability in different age groups: evaluation of clinical outcome measures. Indian J. Orthop. 55 (Suppl. 1), 199–208. 10.1007/s43465-020-00192-6 34122771 PMC8149549

[B2] Alix-Fages C. Romero-Arenas S. Castro-Alonso M. Colomer-Poveda D. Río-Rodriguez D. Jerez-Martínez A. (2019). Short-term effects of anodal transcranial direct current stimulation on endurance and maximal force production. A systematic review and meta-analysis. J. Clin. Med. 8 (4), 536. 10.3390/jcm8040536 31003550 PMC6518246

[B3] Alizamani S. Ghasemi G. Lenjan Nejadian S. (2023). Effects of eight week core stability training on stable- and unstable-surface on ankle muscular strength, proprioception, and dorsiflexion in athletes with chronic ankle instability. J. Bodyw. Mov. Ther. 34, 6–12. 10.1016/j.jbmt.2023.04.005 37301558

[B4] Barr M. J. Sheppard J. M. Agar-Newman D. J. Newton R. U. (2014). Transfer effect of strength and power training to the sprinting kinematics of international rugby players. J. Strength Cond. Res. 28 (9), 2585–2596. 10.1519/jsc.0000000000000423 24552802

[B5] Bouillon L. E. Hofener M. O'Donnel A. Milligan A. Obrock C. (2020). Comparison of muscle activity using unstable devices during a forward lunge. J. Sport Rehabil. 29 (4), 394–399. 10.1123/jsr.2018-0296 30860420

[B6] Bruce A. S. Howard J. S. H V. A. N. W. McBride J. M. Needle A. R. (2020). The effects of transcranial direct current stimulation on chronic ankle instability. Med. Sci. Sports Exerc 52 (2), 335–344. 10.1249/mss.0000000000002129 31453883

[B7] Butcher N. J. Monsour A. Mew E. J. Chan A. W. Moher D. Mayo-Wilson E. (2022). Guidelines for reporting outcomes in trial reports: the CONSORT-outcomes 2022 extension. Jama 328 (22), 2252–2264. 10.1001/jama.2022.21022 36511921

[B8] Cabarkapa D. Cabarkapa D. V. Aleksic J. Philipp N. M. Scott A. A. Johnson Q. R. (2023). Differences in countermovement vertical jump force-time metrics between starting and non-starting professional male basketball players. Front. Sports Act. Living 5, 1327379. 10.3389/fspor.2023.1327379 38162698 PMC10755471

[B9] Claudino J. G. Cronin J. Mezêncio B. McMaster D. T. McGuigan M. Tricoli V. (2017). The countermovement jump to monitor neuromuscular status: a meta-analysis. J. Sci. Med. Sport 20 (4), 397–402. 10.1016/j.jsams.2016.08.011 27663764

[B10] Cohen J. (2016). A power primer. 10.1037//0033-2909.112.1.155 19565683

[B11] Cressey E. M. West C. A. Tiberio D. P. Kraemer W. J. Maresh C. M. (2007). The effects of ten weeks of lower-body unstable surface training on markers of athletic performance. J. Strength Cond. Res. 21 (2), 561–567. 10.1519/r-19845.1 17530966

[B12] Cuğ M. Duncan A. Wikstrom E. (2016). Comparative effects of different balance-training-progression styles on postural control and ankle force production: a randomized controlled trial. J. Athl. Train. 51 (2), 101–110. 10.4085/1062-6050-51.2.08 26878257 PMC4852315

[B13] Delahunt E. Coughlan G. F. Caulfield B. Nightingale E. J. Lin C. W. Hiller C. E. (2010). Inclusion criteria when investigating insufficiencies in chronic ankle instability. Med. Sci. Sports Exerc 42 (11), 2106–2121. 10.1249/MSS.0b013e3181de7a8a 20351590

[B14] de Moura M. Hazime F. A. Marotti Aparicio L. V. Grecco L. A. C. Brunoni A. R. Hasue R. H. (2019). Effects of transcranial direct current stimulation (tDCS) on balance improvement: a systematic review and meta-analysis. Somatosens. Mot. Res. 36 (2), 122–135. 10.1080/08990220.2019.1624517 31181963

[B15] Doherty C. Delahunt E. Caulfield B. Hertel J. Ryan J. Bleakley C. (2014). The incidence and prevalence of ankle sprain injury: a systematic review and meta-analysis of prospective epidemiological studies. Sports Med. 44 (1), 123–140. 10.1007/s40279-013-0102-5 24105612

[B16] Dos'Santos T. McBurnie A. Comfort P. Jones P. A. (2019). The effects of six-weeks change of direction speed and technique modification training on cutting performance and movement quality in male youth soccer players. Sports (Basel) 7 (9), 205. 10.3390/sports7090205 31489929 PMC6783855

[B18] Fong D. T. Hong Y. Chan L. K. Yung P. S. Chan K. M. (2007). A systematic review on ankle injury and ankle sprain in sports. Sports Med. 37 (1), 73–94. 10.2165/00007256-200737010-00006 17190537

[B19] Gibbons T. J. Bird M. L. (2019). Exercising on different unstable surfaces increases core abdominal muscle thickness: an observational study using real-time ultrasound. J. Sport Rehabil. 28 (8), 803–808. 10.1123/jsr.2017-0385 30526226

[B20] Gribble P. A. Bleakley C. M. Caulfield B. M. Docherty C. L. Fourchet F. Fong D. T. (2016). Evidence review for the 2016 International Ankle Consortium consensus statement on the prevalence, impact and long-term consequences of lateral ankle sprains. Br. J. Sports Med. 50 (24), 1496–1505. 10.1136/bjsports-2016-096189 27259753

[B21] Hall E. A. Chomistek A. K. Kingma J. J. Docherty C. L. (2018). Balance- and strength-training protocols to improve chronic ankle instability deficits, Part I: assessing clinical outcome measures. J. Athl. Train. 53 (6), 568–577. 10.4085/1062-6050-385-16 29975573 PMC6089027

[B22] Hall E. A. Docherty C. L. Simon J. Kingma J. J. Klossner J. C. (2015). Strength-training protocols to improve deficits in participants with chronic ankle instability: a randomized controlled trial. J. Athl. Train. 50 (1), 36–44. 10.4085/1062-6050-49.3.71 25365134 PMC4299733

[B23] Han K. Ricard M. D. (2011). Effects of 4 weeks of elastic-resistance training on ankle-evertor strength and latency. J. Sport Rehabil. 20 (2), 157–173. 10.1123/jsr.20.2.157 21576708

[B24] Han S. Son S. J. Kim H. Lee H. Seeley M. Hopkins T. (2022). Prelanding movement strategies among chronic ankle instability, coper, and control subjects. Sports Biomech. 21 (4), 391–407. 10.1080/14763141.2021.1927163 34042012

[B25] Hertel J. Corbett R. O. (2019). An updated model of chronic ankle instability. J. Athl. Train. 54 (6), 572–588. 10.4085/1062-6050-344-18 31162943 PMC6602403

[B26] Holgado D. Zandonai T. Ciria L. F. Zabala M. Hopker J. Sanabria D. (2019). Transcranial direct current stimulation (tDCS) over the left prefrontal cortex does not affect time-trial self-paced cycling performance: evidence from oscillatory brain activity and power output. PLoS One 14 (2), e0210873. 10.1371/journal.pone.0210873 30726234 PMC6364890

[B27] Hu K. Chen Y. Guo F. Wang X. (2022). Effects of transcranial direct current stimulation on upper limb muscle strength and endurance in healthy individuals: a systematic review and meta-analysis. Front. Physiol. 13, 834397. 10.3389/fphys.2022.834397 35356085 PMC8959826

[B28] Huang X. Gao H. Fu H. (2024). Effects of transcranial direct current stimulation combined with Bosu ball training on the injury potential during drop landing in people with chronic ankle instability. Front. Physiol. 15, 1451556. 10.3389/fphys.2024.1451556 39210968 PMC11359566

[B29] Kim H. Son S. J. Seeley M. K. Hopkins J. T. (2019). Altered movement strategies during jump landing/cutting in patients with chronic ankle instability. Scand. J. Med. Sci. Sports 29 (8), 1130–1140. 10.1111/sms.13445 31050053

[B30] Kohler J. M. Flanagan S. P. Whiting W. C. (2010). Muscle activation patterns while lifting stable and unstable loads on stable and unstable surfaces. J. Strength Cond. Res. 24 (2), 313–321. 10.1519/JSC.0b013e3181c8655a 20072068

[B31] Konradsen L. Bech L. Ehrenbjerg M. Nickelsen T. (2002). Seven years follow-up after ankle inversion trauma. Scand. J. Med. Sci. Sports 12 (3), 129–135. 10.1034/j.1600-0838.2002.02104.x 12135444

[B32] Kristianslund E. Bahr R. Krosshaug T. (2011). Kinematics and kinetics of an accidental lateral ankle sprain. J. Biomech. 44 (14), 2576–2578. 10.1016/j.jbiomech.2011.07.014 21824618

[B33] Li Y. Ko J. Zhang S. Brown C. N. Simpson K. J. (2019). Biomechanics of ankle giving way: a case report of accidental ankle giving way during the drop landing test. J. Sport Health Sci. 8 (5), 494–502. 10.1016/j.jshs.2018.01.002 31534824 PMC6742755

[B34] Li Y. Wang Z. Shen Y. Yang Y. Wang X. Liu H. (2024). Differences in cortical activation during dorsiflexion and plantarflexion in chronic ankle instability: a task-fMRI study. Clin. Orthop. Relat. Res. 482 (5), 814–826. 10.1097/corr.0000000000002903 37938129 PMC11008668

[B35] Lis D. M. Jordan M. Lipuma T. Smith T. Schaal K. Baar K. (2022). Collagen and vitamin C supplementation increases lower limb rate of force development. Int. J. Sport Nutr. Exerc Metab. 32 (2), 65–73. 10.1123/ijsnem.2020-0313 34808597

[B36] Liu Y. Dong S. Wang Q. Liu Z. Song Q. Shen P. (2024). Deficits in proprioception and strength may contribute to the impaired postural stability among individuals with functional ankle instability. Front. Physiol. 15, 1342636. 10.3389/fphys.2024.1342636 38496300 PMC10941841

[B37] Ma Y. Yin K. Zhuang W. Zhang C. Jiang Y. Huang J. (2020). Effects of combining high-definition transcranial direct current stimulation with short-foot exercise on chronic ankle instability: a pilot randomized and double-blinded study. Brain Sci. 10 (10), 749. 10.3390/brainsci10100749 33080863 PMC7602979

[B38] Madhavan S. Shah B. (2012). Enhancing motor skill learning with transcranial direct current stimulation - a concise review with applications to stroke. Front. Psychiatry 3, 66. 10.3389/fpsyt.2012.00066 22807918 PMC3395020

[B39] McKay G. D. Goldie P. A. Payne W. R. Oakes B. W. (2001). Ankle injuries in basketball: injury rate and risk factors. Br. J. Sports Med. 35 (2), 103–108. 10.1136/bjsm.35.2.103 11273971 PMC1724316

[B40] Meeuwisse W. H. (1991). Predictability of sports injuries. What is the epidemiological evidence? Sports Med. 12 (1), 8–15. 10.2165/00007256-199112010-00002 1925190

[B41] Morin J. B. Slawinski J. Dorel S. de Villareal E. S. Couturier A. Samozino P. (2015). Acceleration capability in elite sprinters and ground impulse: push more, brake less? J. Biomech. 48 (12), 3149–3154. 10.1016/j.jbiomech.2015.07.009 26209876

[B42] Muellbacher W. Ziemann U. Wissel J. Dang N. Kofler M. Facchini S. (2002). Early consolidation in human primary motor cortex. Nature 415 (6872), 640–644. 10.1038/nature712 11807497

[B43] Nam H. C. Cha H. G. Kim M. K. (2016). The effects of exercising on an unstable surface on the gait and balance ability of normal adults. J. Phys. Ther. Sci. 28 (7), 2102–2104. 10.1589/jpts.28.2102 27512275 PMC4968516

[B44] Nanbancha A. Tretriluxana J. Limroongreungrat W. Sinsurin K. (2019). Decreased supraspinal control and neuromuscular function controlling the ankle joint in athletes with chronic ankle instability. Eur. J. Appl. Physiol. 119 (9), 2041–2052. 10.1007/s00421-019-04191-w 31321512

[B45] Needle A. R. Lepley A. S. Grooms D. R. (2017). Central nervous system adaptation after ligamentous injury: a summary of theories, evidence, and clinical interpretation. Sports Med. 47 (7), 1271–1288. 10.1007/s40279-016-0666-y 28005191

[B46] Nigg B. M. Stefanyshyn D. J. Rozitis A. I. Mündermann A. (2009). Resultant knee joint moments for lateral movement tasks on sliding and non-sliding sport surfaces. J. sports Sci. 27 (5), 427–435. 10.1080/02640410802669161 19253080

[B47] Nitsche M. A. Liebetanz D. Lang N. Antal A. Tergau F. Paulus W. (2003). Safety criteria for transcranial direct current stimulation (tDCS) in humans. Clin. Neurophysiol. 114 (11), 2220–2222. 10.1016/s1388-2457(03)00235-9 14580622

[B48] O'Connor K. M. Bottum M. C. (2009). Differences in cutting knee mechanics based on principal components analysis. Med. Sci. Sports Exerc 41 (4), 867–878. 10.1249/MSS.0b013e31818f8743 19276846

[B49] Parenteau C. S. Viano D. C. Petit P. Y. (1998). Biomechanical properties of human cadaveric ankle-subtalar joints in quasi-static loading. J. Biomech. Eng. 120 (1), 105–111. 10.1115/1.2834289 9675688

[B50] Paulus W. (2003). Transcranial direct current stimulation (tDCS). Suppl. Clin. Neurophysiology 56 (35), 249–254. 10.1016/s1567-424x(09)70229-6 14677402

[B51] Peterson M. D. Alvar B. A. Rhea M. R. (2006). The contribution of maximal force production to explosive movement among young collegiate athletes. J. Strength Cond. Res. 20 (4), 867–873. 10.1519/r-18695.1 17194245

[B17] Pierce Ca B. R. Aguinis H. (2004). Cautionary note on reporting eta-squared values from multifactor ANOVA designs. Educ. Psychol. Meas. 64 (6), 916–924. 10.1177/0013164404264848

[B52] Pietrosimone B. G. Gribble P. A. (2012). Chronic ankle instability and corticomotor excitability of the fibularis longus muscle. J. Athl. Train. 47 (6), 621–626. 10.4085/1062-6050-47.6.11 23182009 PMC3499885

[B53] Pirauá A. L. T. Cavalcante B. R. de Oliveira V. M. A. Beltrão N. B. de Amorim Batista G. Pitangui A. C. R. (2019). Effect of 24-week strength training on unstable surfaces on mobility, balance, and concern about falling in older adults. Scand. J. Med. Sci. Sports 29 (11), 1805–1812. 10.1111/sms.13510 31273863

[B54] Reis J. Fritsch B. (2011). Modulation of motor performance and motor learning by transcranial direct current stimulation. Curr. Opin. Neurol. 24 (6), 590–596. 10.1097/WCO.0b013e32834c3db0 21968548

[B55] Reis J. Schambra H. M. Cohen L. G. Buch E. R. Fritsch B. Zarahn E. (2009). Noninvasive cortical stimulation enhances motor skill acquisition over multiple days through an effect on consolidation. Proc. Natl. Acad. Sci. U. S. A. 106 (5), 1590–1595. 10.1073/pnas.0805413106 19164589 PMC2635787

[B56] Romero-Arenas S. Calderón-Nadal G. Alix-Fages C. Jerez-Martínez A. Colomer-Poveda D. Márquez G. (2021). Transcranial direct current stimulation does not improve countermovement jump performance in young healthy men. J. Strength Cond. Res. 35 (10), 2918–2921. 10.1519/jsc.0000000000003242 31373982

[B57] Rosen A. B. Yentes J. M. McGrath M. L. Maerlender A. C. Myers S. A. Mukherjee M. (2019). Alterations in cortical activation among individuals with chronic ankle instability during single-limb postural control. J. Athl. Train. 54 (6), 718–726. 10.4085/1062-6050-448-17 31162942 PMC6602391

[B58] Sepasgozar Sarkhosh S. Khanmohammadi R. Shiravi Z. (2024). Comparison of the effects of exergaming and balance training on dynamic postural stability during jump-landing in recreational athletes with chronic ankle instability. PLoS One 19 (12), e0314686. 10.1371/journal.pone.0314686 39680600 PMC11649137

[B59] Shyamali Kaushalya F. Romero-Arenas S. García-Ramos A. Colomer-Poveda D. Marquez G. (2022). Acute effects of transcranial direct current stimulation on cycling and running performance. A systematic review and meta-analysis. Eur. J. Sport Sci. 22 (2), 113–125. 10.1080/17461391.2020.1856933 33280514

[B60] Simpson J. D. Koldenhoven R. M. Wilson S. J. Stewart E. M. Turner A. J. Chander H. (2020). Ankle kinematics, center of pressure progression, and lower extremity muscle activity during a side-cutting task in participants with and without chronic ankle instability. J. Electromyogr. Kinesiol 54, 102454. 10.1016/j.jelekin.2020.102454 32777448

[B61] Stagg C. J. Jayaram G. Pastor D. Kincses Z. T. Matthews P. M. Johansen-Berg H. (2011). Polarity and timing-dependent effects of transcranial direct current stimulation in explicit motor learning. Neuropsychologia 49 (5), 800–804. 10.1016/j.neuropsychologia.2011.02.009 21335013 PMC3083512

[B62] Stagg C. J. Nitsche M. A. (2011). Physiological basis of transcranial direct current stimulation. Neuroscientist 17 (1), 37–53. 10.1177/1073858410386614 21343407

[B63] Suchomel T. J. Nimphius S. Stone M. H. (2016). The importance of muscular strength in athletic performance. Sports Med. 46 (10), 1419–1449. 10.1007/s40279-016-0486-0 26838985

[B64] Talimkhani A. Abdollahi I. Mohseni-Bandpei M. A. Ehsani F. Khalili S. Jaberzadeh S. (2019). Differential effects of unihemispheric concurrent dual-site and conventional tDCS on motor learning: a randomized, sham-controlled study. Basic Clin. Neurosci. 10 (1), 59–72. 10.32598/bcn.9.10.350 31031894 PMC6484181

[B65] Tashiro T. Maeda N. Sasadai J. Kotoshiba S. Sakai S. Suzuki Y. (2021). Tensiomyographic neuromuscular response of the peroneus longus and tibialis anterior with chronic ankle instability. Healthc. (Basel) 9 (6), 707. 10.3390/healthcare9060707 PMC8230383 34200684

[B66] Terada M. Pietrosimone B. G. Gribble P. A. (2014). Alterations in neuromuscular control at the knee in individuals with chronic ankle instability. J. Athl. Train. 49 (5), 599–607. 10.4085/1062-6050-49.3.28 25144597 PMC4208863

[B67] Tropp H. (2002). Commentary: functional ankle instability revisited. J. Athl. Train. 37 (4), 512–515. 12937576 PMC164386

[B68] Verhagen E. van der Beek A. Twisk J. Bouter L. Bahr R. van Mechelen W. (2004). The effect of a proprioceptive balance board training program for the prevention of ankle sprains: a prospective controlled trial. Am. J. Sports Med. 32 (6), 1385–1393. 10.1177/0363546503262177 15310562

[B69] Villamar M. F. Volz M. S. Bikson M. Datta A. Dasilva A. F. Fregni F. (2013). Technique and considerations in the use of 4x1 ring high-definition transcranial direct current stimulation (HD-tDCS). J. Vis. Exp. 77, e50309. 10.3791/50309 PMC3735368 23893039

[B70] Wang B. Zhang X. Zhu F. Zhu W. Wang X. Jia F. (2022). A randomized controlled trial comparing rehabilitation with isokinetic exercises and Thera-Band strength training in patients with functional ankle instability. PLoS One 17 (12), e0278284. 10.1371/journal.pone.0278284 36454876 PMC9714719

[B71] Ward S. Pearce A. J. Pietrosimone B. Bennell K. Clark R. Bryant A. L. (2015). Neuromuscular deficits after peripheral joint injury: a neurophysiological hypothesis. Muscle Nerve 51 (3), 327–332. 10.1002/mus.24463 25255714

[B72] Wester J. U. Jespersen S. M. Nielsen K. D. Neumann L. (1996). Wobble board training after partial sprains of the lateral ligaments of the ankle: a prospective randomized study. J. Orthop. Sports Phys. Ther. 23 (5), 332–336. 10.2519/jospt.1996.23.5.332 8728532

[B73] Whitting J. W. de Melker Worms J. L. Maurer C. Nigg S. R. Nigg B. M. (2013). Measuring lateral shuffle and side cut performance. J. Strength Cond. Res. 27 (11), 3197–3203. 10.1519/JSC.0b013e31828a2c2b 23439340

[B74] Xue X. Ma T. Li Q. Song Y. Hua Y. (2021). Chronic ankle instability is associated with proprioception deficits: a systematic review and meta-analysis. J. Sport Health Sci. 10 (2), 182–191. 10.1016/j.jshs.2020.09.014 33017672 PMC7987558

[B75] Zhang J. Yang K. Wang C. Gu W. Li X. Fu S. (2023). Risk factors for chronic ankle instability after first episode of lateral ankle sprain: a retrospective analysis of 362 cases. J. Sport Health Sci. 12 (5), 606–612. 10.1016/j.jshs.2023.03.005 36931594 PMC10466191

[B76] ZhuX.WeiF.LiS.ZhangT.ShenP.FongD. T. (2025). Toe-out landing reduces anterior talofibular ligament strain while maintains calcaneofibular ligament strain in people with chronic ankle instability. J. Sport Health Sci., 101035. 10.1016/j.jshs.2025.101035 40021056

